# Dynamic games for secure and resilient control system design

**DOI:** 10.1093/nsr/nwz218

**Published:** 2020-01-16

**Authors:** Yunhan Huang, Juntao Chen, Linan Huang, Quanyan Zhu

**Affiliations:** Department of Electrical and Computer Engineering, New York University, Brooklyn, NY 11201, USA; Department of Electrical and Computer Engineering, New York University, Brooklyn, NY 11201, USA; Department of Electrical and Computer Engineering, New York University, Brooklyn, NY 11201, USA; Department of Electrical and Computer Engineering, New York University, Brooklyn, NY 11201, USA

**Keywords:** dynamic games, robustness, security, resilience, cyber-physical system, complex systems

## Abstract

Modern control systems are featured by their hierarchical structure composed of cyber, physical and human layers. The intricate dependencies among multiple layers and units of modern control systems require an integrated framework to address cross-layer design issues related to security and resilience challenges. To this end, game theory provides a bottom-up modeling paradigm to capture the strategic interactions among multiple components of the complex system and enables a holistic view to understand and design cyber-physical-human control systems. In this review, we first provide a multi-layer perspective toward increasingly complex and integrated control systems and then introduce several variants of dynamic games for modeling different layers of control systems. We present game-theoretic methods for understanding the fundamental tradeoffs of robustness, security and resilience and developing a cross-layer approach to enhance the system performance in various adversarial environments. This review also includes three quintessential research problems that represent three research directions where dynamic game approaches can bridge between multiple research areas and make significant contributions to the design of modern control systems. The paper is concluded with a discussion on emerging areas of research that crosscut dynamic games and control systems.

## INTRODUCTION

**Figure 1.**
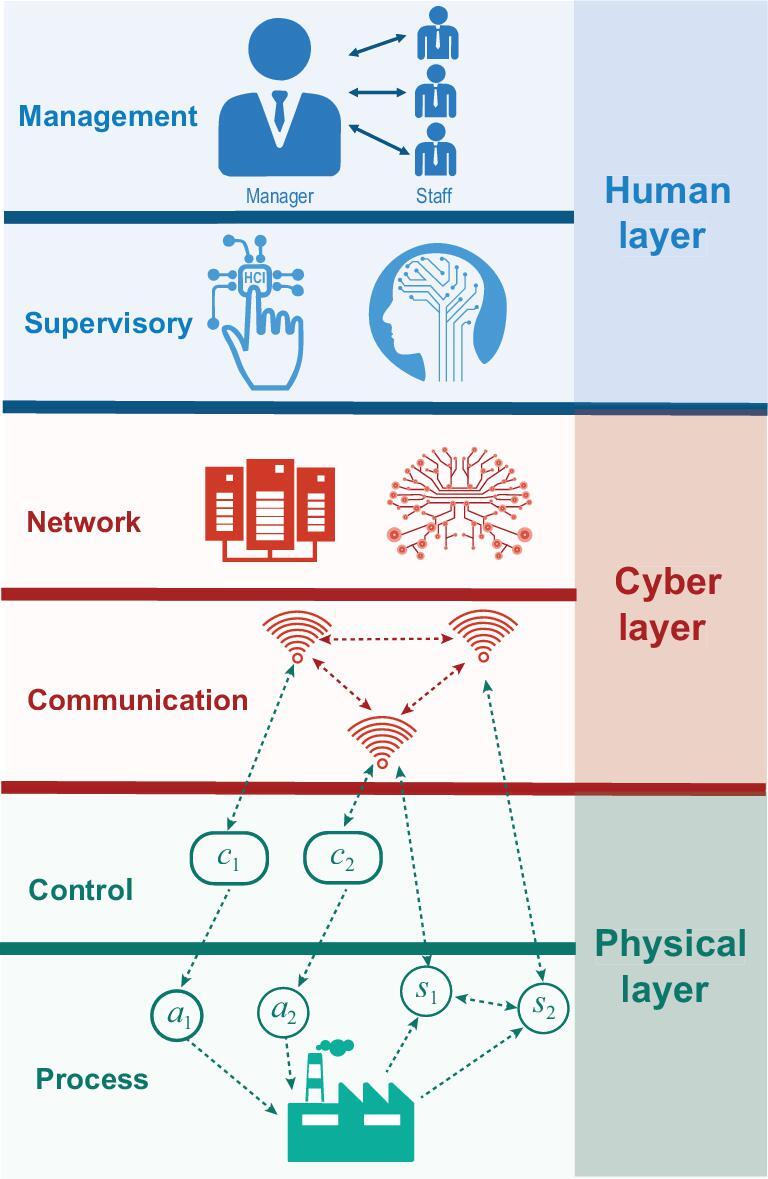
The hierarchical structure of modern control systems is composed of six layers. The physical layer consists of a physical plant embedded with actuators and sensors. The control system receives orders, observations and sends commands to actuators to achieve desired system performance. The communication layer provides wired or wireless data communications that enable advanced monitoring and intelligent control. The network layer allocates network resources for routing and provides interconnections between system units. The supervisory layer serves as the executive brain of the entire system, provides human–machine interactions, and coordinates and manages lower layers through centralized command and control. The management layer resides at the highest echelon. It deals with social and economic issues, such as market regulation, pricing, incentive and environmental affairs.

Recent advances in information and communications technologies (ICTs) such as the Internet of Things (IoT) and 5G high-speed networks have witnessed increasing connectivity between control systems and cyber networks. The integration between the cyber and physical worlds has made significant advances in many industrial sectors and critical infrastructures, including electric power, manufacturing and transportation, heralding the fourth industrial revolution that transforms the operation of industrial control systems. To understand and design such systems would require a global and hierarchical perspective toward modern control systems as shown in Fig. 1. The classical view toward control systems consists of sensing, control and plant dynamics integrated in a feedback loop.

A multitude of control design methods including robust control, adaptive control and stochastic control have focused on how to deal with uncertainties and physical disturbances [1]. Modern control systems, due to their exposure to open networks and integration with complex software, require new methodologies that go beyond the classical ones that have focused on the interface between the control layer and the plant at the physical layer. The classical control system is extended by interconnecting it with the cyber and human layers. The cyber layer consists of the communication and networking issues that arise from the communications between sensors and actuators as well as the connectivity among multiple distributed agents. The human layer consists of the supervisory and the management layers that deal with the issues that include coordination, operation, planning and investment.

As the modern control system design benefits from the growing connectivity, the innate vulnerabilities at the cyber layer and the human layer in modern control systems can bring concomitant threats and hazards from adversaries [2]. Many incidents have been reported as a result of attacker’s exploitation of these vulnerabilities [3,4]. Stuxnet, reported in Refs [5,6], is one of the well-known Advanced Persistent Threats (APTs) to control systems that can persist for a long period, behave stealthily and specifically target industrial control systems by taking advantage of the Supervisory Control And Data Acquisition (SCADA) systems. This type of attack can also be launched by an insider. One example is the Maroochy water breach incident launched by a disgruntled former employee. The attack surface of control systems is exponentially growing. Adversaries can exploit multiple zero-day vulnerabilities and launch unanticipated attacks. One example is the recent hacking of the self-driving vehicles, where the attacker has remotely manipulated, through the cellular connection of the vehicle, various electronic control units, from wiper to brake and engine system [7]. Apart from self-driving vehicles, many other autonomous systems can face similar threats. Failure to defend against such threats can inflict huge financial losses and fatal damages.

The adversarial behaviors at the human and the cyber layers are often hard to anticipate and prepare for. They can cause a significant amount of catastrophic damage to control systems in terms of their high impact and low effort. The classical approach that regards abnormal behaviors as a result of uncertainties and perturbations to physical plants is insufficient to address these emerging threats. To this end, a new design paradigm is needed to develop frameworks to safeguard the control systems from cyber threats and mitigate the damage that can be caused by attacks. In other words, it is indispensable to consider system properties beyond stability and establish a holistic framework to incorporate the study of robustness, security and resilience of control systems.

This review aims to present an extensive overview of recent research directions on using game-theoretic approaches to address robust, secure and resilient design problems of modern control systems. The first objective of this review is to provide a layering perspective toward modern control systems that consist of cyber, physical and human components across the layers. Game-theoretic methods play an important role in interconnecting different aspects of a control system and providing a holistic and integrated framework to address the cross-layer design of robust, secure and resilient systems. The second objective of this review is to bridge the classical system design approaches and the modern system design through game-theoretic methods. We can view the secure and resilient control design as an extension of the classical robust control design by integrating multiple game-theoretic frameworks. Last but not least, the third objective of this work is to introduce the emerging research topics related to game-theoretic methods for secure and resilient control system design. Namely, we present three major application areas including secure and resilient control of heterogeneous autonomous systems, defensive deception games for industrial control systems and risk management of cyber-physical networks. In this review, we focus on game-theoretic methods for a robust, secure and resilient control system design with an emphasis on dynamic games. For game-theoretic security surveys in general Cyber-Physical Systems (CPSs), one can refer to Refs [8–10].

### The triplet: robustness, security and resilience

Robustness, security and resilience are three major control system properties for modern control systems. The notion of robustness describes a system’s ability to maintain its performance in the presence of regular and singular perturbations [11], whereas security refers to the system’s ability to withstand and be protected from malicious behaviors and unanticipated events [1]. Robustness and security are two system properties that are achieved offline by foreseeing the perturbations and the attacks before they happen. Thus, these two system properties are classified as pre-event concepts. Despite many endeavors toward designing robust and secure systems, it is impractical and economically inefficient, if it is possible, to achieve perfect robustness and security against all possible perturbations, attacks and events. This concern calls for the notion of resilience, a post-event concept referring to the system’s ability to recover online after adversarial events occur. Hence, resilient control systems have performance guarantees so that even when robustness and security fail under unanticipated attacks and failures, the systems can self-recover from deterioration.

It is imperative to be aware that robustness, security and resilience are three interdependent concepts. These three system properties should be jointly considered in the design of modern control systems. Since a robust control system can withstand a certain range of uncertain parameters and disturbances, the system stays safe under the malicious attacks if the design of security can limit the impact of the malicious attacks within an acceptable range. Additionally, the design of resilient control systems pivots on the fundamental system tradeoffs between robustness, security and resilience. Perfect security could be attained by making the system unusable and likewise, perfect robustness could be reached by considerably degenerating the control performance. The fact that no desirable control systems exhibit perfect robustness or security creates a serious need for resilience. Hence, the three system properties should be jointly designed. It is of vital importance to know, on the one hand, what type of uncertainties or adversarial events need to be considered for enhancing robustness and security, and on the other hand, what uncertainties or malicious events need to be considered for post-event resilience.

**Figure 2.**
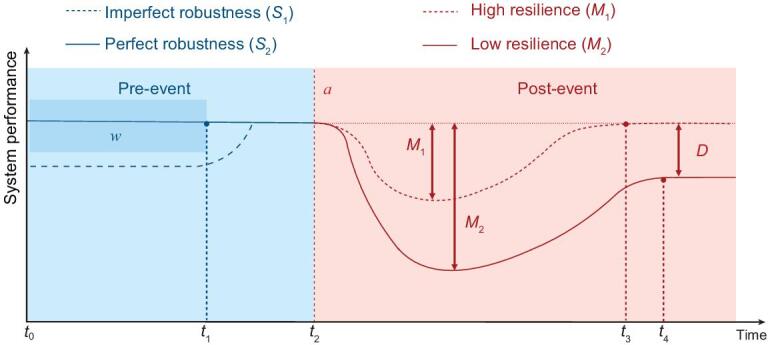
System performance evolves as different events happen. The solid line represents system *S*_1_ while the dashed line represents system *S*_2_. Before *t*_1_, a known small range of disturbances $w$ hits the system. At *t*_2_, an attack or rare event ${\mathit a}$ happens. At *t*_3_, system *S*_1_ finishes full recovery; later at *t*_4_, system *S*_2_ finishes recovery. System *S*_2_ fails to accomplish full recovery and suffers from a steady-state functionality degradation *D*. The maximum functionality degradation of system *S*_1_ (or resp. *S*_2_) induced by the event is denoted by *M*_1_ (or resp. *M*_2_).

Metrics for robustness in control systems have been well established in the literature [11,12]. A game-theoretic approach has been introduced to obtain the *H*^∞^ optimal, disturbance-attenuating minimax controllers by viewing the controller as the cost minimizer and the disturbance as the maximizer. Likewise, game-theoretic frameworks have been established to capture the conflict of goals between an attacker who seeks to escalate the damage inflicted on the system and a defender who aims to mitigate it [13]. There is a rich literature on defining metrics for the security [13–15]. However, metrics for security, unlike those for robustness, are problem dependent as the attack model varies and the security design parameters depend on the defense mechanisms such as cryptography, detection, network architecture and communication protocols. Examples of recent security metrics can be found in Refs [16–20]. Metrics for resilience naturally require a comparison between the pre-event and the post-event performance as resilience is a system property defined as the ability to recover from severe stresses induced by natural disasters or malicious attacks. Figure 2 illustrates the notion of resilience with respect to an attack that is launched at time *t*_1_. Shortly after the attack, the system performance starts to degrade to its maximum degree *M*_1_ and *M*_2_ for the high-resiliency system (*S*_2_) and the low-resiliency system (*S*_1_), respectively. Recovery mechanisms are used to restore the system to its original performance or a steady-state degraded performance for system *S*_2_ and *S*_1_, respectively. A system is said to be more resilient if the system is capable of recovering after an attack with a lower loss of performance and a faster recovery time. The most commonly used mathematical definition of resilience is provided in Refs [16,21].

### Game-theoretic methods

Game theory [22,23], in a nutshell, studies the strategic interaction between two or multiple decision-makers, called players, where each player aims to optimize his respective objective function, which depends on the choices of other players in the game. Hence, the optimal decisions of the players are coupled when they aim to achieve the best for themselves. Game theory provides a powerful modeling tool to describe strategic interactions among players. Based on objectives of the players, games can be divided into two categories: zero-sum games and non-zero-sum games.

A zero-sum game refers to a two-player game where the sum of the two players’ objective functions is zero or can be made zero by appropriate positive scaling and/or translation that do not depend on the decision variables of the player. Zero-sum games are often used to describe conflicting objectives between two players where one player’s gain is the other player’s loss. Security games often take the form of zero-sum games as in Blotto games [24] and adversarial machine learning problems [25]. A non-cooperative game is non-zero-sum if the sum of the players’ objective functions cannot be made zero. If each player in a game has only a finite number of alternatives, this game is finite, or a matrix game; otherwise, it is an infinite game. A continuous-kernel game is an infinite game where the action sets of the players are subsets of finite-dimensional vector spaces, and the players’ objective functions are continuous with respect to the action variables of all players. A game is dynamic when players interact multiple rounds sequentially. A game is of complete information if the structure of the game being played is of common information to all players, including the number of players, the objective functions of the players, the underlying dynamics, the information structure, etc.; it is of incomplete information otherwise.

The concepts of equilibrium play a vital role in game theory which refers to a joint strategy profile from which no player has a unilateral incentive to change his strategy within the rules of the game. Based on the types of game, we have various notions of equilibrium including the Nash equilibrium, Stackelberg equilibrium, saddle-point equilibrium (SPE), Bayesian equilibrium, etc. They are useful to describe the outcomes of different types of interactions among players. For a detailed exposition of basic concepts of equilibrium solutions, we refer the reader to Refs [22,23]; and for a review of game-theoretic applications to cyber security, we refer readers to Refs [13,26–28].

Dynamic games are useful to model multi-layer interactions in control systems as the system dynamics evolve, and different components across the players contribute to the path of the dynamics. For example, the adversary who disrupts the communication channels can create a denial-of-service attack that makes sensor data unavailable and hence leads the plant dynamics toward an unstable trajectory. The negligence of a human operator can expose the control system network to malware, which aims to disrupt the normal operations of a nuclear power plant. In dynamic games, the information structure of the game, the form of dynamical systems, and the constraints on the strategy space determine different classes of dynamic game models that are useful to describe a rich class of scenarios of interactions for control systems. For example, the design of robust control systems has been successfully formulated as a continuous-time differential game between disturbance and controller, which are regarded as two players [11,12,17,23]. The controller seeks to minimize the control cost criterion by choosing a controller that adapts to a given information structure while the disturbance aims to maximize it.

The design of security mechanisms against APT attacks can be viewed as a multi-stage game where an attacker aims to find a path toward the control system network from its initial entry point while the network defender aims to detect and deter the attack from reaching the targeted asset [29–31]. If the attacker is prevented from reaching the objective or removed from the system, the system is successfully defended. However, when the network defender fails to safeguard the control system from the attack, the resilience strategies need to be planned to restore the attacked control system to its original operation. Resiliency should be built on the robustness and the security of the system as the post-event resiliency relies on the pre-event designs [32–37].

Hence, the pre-event secure strategy and the post-event resilience strategy are designed as a result of the game between the defender and the attacker. Despite the fact that security games are structurally different from robust control games and may take different forms depending on attack models [1,13,20,24,38–48], both security/resilience and robustness of control systems can be studied using dynamic game frameworks. Thus, dynamic games provide a holistic approach to create an integrated framework to design robust, secure and resilient control systems by composing different types of games together, as shown in recent literature [e.g. 1,16–20,39,49,50].

## 2 DYNAMIC GAMES FOR ROBUSTNESS, SECURITY AND RESILIENCE

Modern control systems primarily consist of six layers: physical, control, communication, network, supervisory and management, as illustrated in Fig. 1. Sitting at the bottom is the physical world of the system which serves as a foundation for modern control systems. The physical world of the system can be viewed as an integration of the physical plant to be controlled and the control layer providing control signals based on the feedback. On top of these two layers are the communication layer, which establishes wired or wireless communications, and the network layer, which allocates resources and manages routing. The communication and network layers constitute the cyber world of the system. Note that in remote control systems, the control layer can be sitting above the cyber layer. Systems containing mainly the cyber layer and the physical layer are called cyber-physical systems. Serving as the brain of the system, the supervisory layer coordinates the cyber and physical layers by designing and sending appropriate commands. Together with the supervisory layer, the management layer interfaces with humans and makes high-level decisions, creating a human-in-the-loop cyber-physical control system.

The design of the cyber-physical control system used to be a compartmentalized process, where the cyber system engineers design network protocols and security policies independent from the engineers who design control laws for the underlying physical or chemical processes. This practice, however, is not sufficient to meet the integrated system requirements when the two systems are tightly coupled and strongly interdependent. It is imperative to take into account cyber security when designing control laws for the physical systems, and be aware of the physical impact when designing communications protocols and configuring network devices.

### The cyber-physical-human system framework

**Figure 3.**
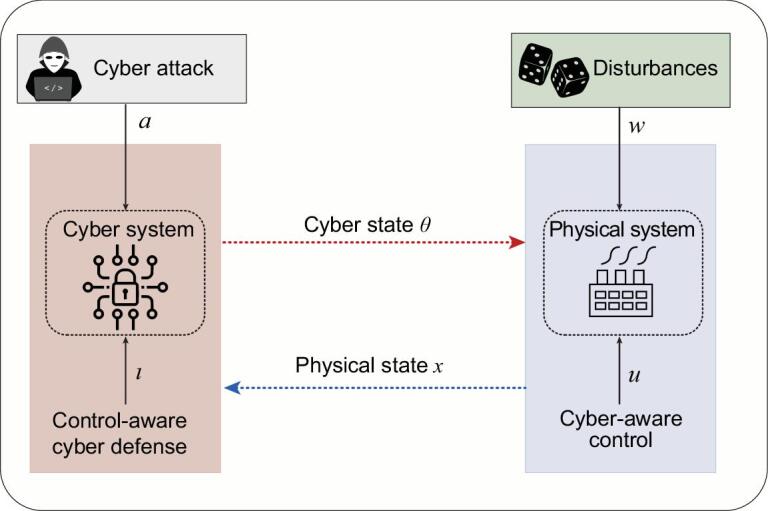
Illustration of the security-aware resilient control systems.

The baseline security-aware resilient control systems are illustrated in Fig. 3, and can be mathematically described using the following dynamical system model:

(1) \begin{eqnarray*} \dot{\mathbf {x}}(t) = f(t, \mathbf {x}, \mathbf {u}, \mathbf {w}; \theta (t, a, l)), \ \ \mathbf {x}(t_0)=x_0, \end{eqnarray*}

(2) \begin{eqnarray*} \mathbf {y}(t) = h(t, \mathbf {x}, {\mathbf {u}}, \mathbf {w}; \theta (t,a,l)), \end{eqnarray*}

where *f* and *h* are continuous functions in $(t,\mathbf {x}, \mathbf {u},\mathbf {w})$; $\mathbf {x}(t)\in \mathbb {R}^n$ is the state of the physical system; $\mathbf {y}(t) \in \mathbb {R}^m$ is the sensor measurement; *x*_0_ is a fixed (known) initial state of the physical plant at starting time *t*_0_; $\mathbf {u}(t)\in \mathbb {R}^r$ is the control input; $\mathbf {w}(t)$ models the combined disturbances on the plant and the sensors. The effect of higher layers on the physical layer is encoded in θ which could be a function of time. The space that θ lies in is problem-dependent. The evolution of θ depends on the cyber defense action *l* and the attacker’s action *a*, which could also be functions of time. We use θ(*t*) as a shorthand notation in place of θ(*t, a, l*) if the pair of actions (*a, l*) is fixed.

#### Cyber attack and defense

For example, given pair (*a, l*), θ(*t*), *t* ∈ [0, *t*_*f*_], could be a Markov jump process with right-continuous sample paths, with initial distribution π_0_, and with rate matrix $\lambda =\lbrace \lambda _{ij}\rbrace _{i,j\in \mathcal {S}}$, where $\mathcal {S}:=\lbrace 1, 2, \cdots , s\rbrace$ is the state space; $\lambda _{ij} \in \mathbb {R}_{+}$ are the transition rates such that for *i* ≠ *j*, λ_*ij*_ ≥ 0 and λ_*ii*_ = 1 − ∑_*j* ≠ *i*_λ_*ij*_ for $i\in \mathcal {S}$.

**Figure 4.**
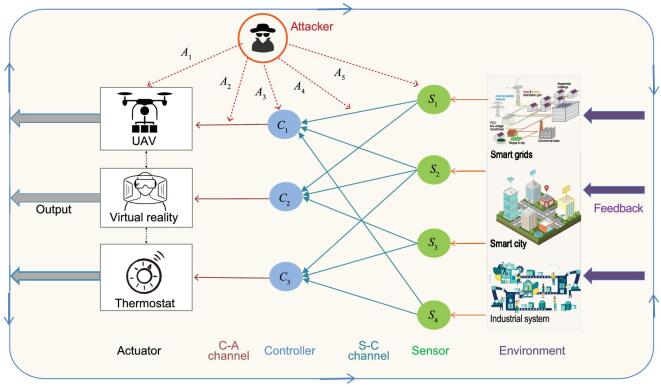
The vulnerabilities of control systems to multiple potential attacks. The attacker can compromise various components in a control system, including sensors, communication channels, controllers and actuators.

The framework can be used to capture different types of attacks on control systems, such as the the replay attack [51,52], the false data injection attack [53], and the sensor attack [54].

(i) In the replay attack, the attacker can record sensor measurements, choose the replay window size *T*_*R*_ > 0 and decide whether to send the original or modified sensor outputs at each time step. Let θ = θ_1_ denote the state of the cyber state where there is no attack, and the control system is in a healthy state. Let θ = θ_2_ denote the state where an attack has been successfully launched in the cyber layer, and the control system is compromised. The replay attack can be captured by letting $h(t, \mathbf {x}, \mathbf {w}; \theta _2) = \mathbf {y}({t-T_R})$ in Eq. (2), stating that the past measurements $\mathbf {y}({t-T_R})$ are taken as the current ones $\mathbf {y}(t)$.
(ii) In the false data injection attack where the attacker injects data to a subset of sensors, the model (2) can be used to capture the attack by letting $h(t, \mathbf {x}, \mathbf {w}; \theta _2) = h(t, \mathbf {x}, \mathbf {w}; \theta _1) + \mathbf {y^a}(t)$, where $\mathbf {y^a}(t)$ is the data value injected by the attacker. In cases where an attacker can cause disruptions to the system operation, for example, by opening a valve in water distribution systems [55], or turning on a circuit breaker in electric power systems [56], the dynamics of the control system will be changed, and they can be captured in Eq. (1) by specifying the changed post-attack dynamics. Figure 4 illustrates the vulnerabilities of control systems to multiple potential attacks, where the controller-actuator (C-A) channel and the sensor-controller (S-C) channels are vulnerable to cyber attacks. *A*_5_ represents direct sensor attacks that can disable a set of sensors or make them send false information to controllers. *A*_3_ and *A*_1_ represent the denial of service (DoS) attacks that prevent controllers from receiving sensor measurements or actuators from receiving control signals. *A*_4_ and *A*_2_ represent data injection attacks on the communication channels, where the false information $\mathbf {\tilde{y}}\ne \mathbf {y}$ and $\mathbf {\tilde{u}}\ne \mathbf {u}$ is sent from sensors and controllers.
(iii) In sensor attacks, θ(*t*) can describe the set of sensors whose signals cannot be received by the control center due to network failure or sensor failure caused by DoS attacks. Each sensor has two states: functioning normally or not. If the number of sensors in the physical plant is *N*, then $\theta (t)\in \mathcal {S}$ and $\mathcal {S}=\lbrace 1,...,2^N\rbrace$. At time *t*, the cyber attack action *a*(*t*) will be to choose a set of sensors to attack and the cyber defense move *l*(*t*) will be to recover a chosen set of sensors. Then, $\lbrace \theta (t)\rbrace _{t\in [0,t_f]}$ becomes a controlled Markov jump process with transition rate ${\lambda _{ij}(a,l)},i,j\in \mathcal {S}$. In this case, the system dynamics is considered to be independent from θ, i.e. $f(t,\mathbf {x},\mathbf {u},\mathbf {w};\theta (t,a,l))=f(t,\mathbf {x},\mathbf {u},\mathbf {w})$. The output *y* is captured by (2). For linear system models, we have $\mathbf {y}=C(\theta (t,a,l)) \mathbf {x}$ where matrix *C* is a function of θ(*t*) decided by the set of sensors that function normally. With different θ, the system designer needs to adapt different schemes to do filtering and control.

The costs of launching attacks and executing defenses are captured by *C*_*A*_(*a, l*) and *C*_*D*_(*a, l*), respectively. The attacker aims to minimize the cost of attacking and deteriorating system performance. Adversely, the system operator aims to minimize the cost of defending and maintaining system performance. If *C*_*A*_(*a, l*) + *C*_*D*_(*a, l*) = 0, the attack-and-defense problem is a zero-sum stochastic game [57] with a Markov decision process sitting behind. In general, we have *C*_*A*_(*a, l*) + *C*_*D*_(*a, l*) ≠ 0. The costs *C*_*A*_ and *C*_*D*_ depend on the attacker’s and the system’s actions and the system performance encoded in **x** while the evolution of **x** is dependent on **u** and θ. Thus, the security and resilience design in the cyber layer is coupled with the system dynamics in the physical plant which should be jointly considered.

#### Robustness and resilience in the physical layer

**Figure 5.**
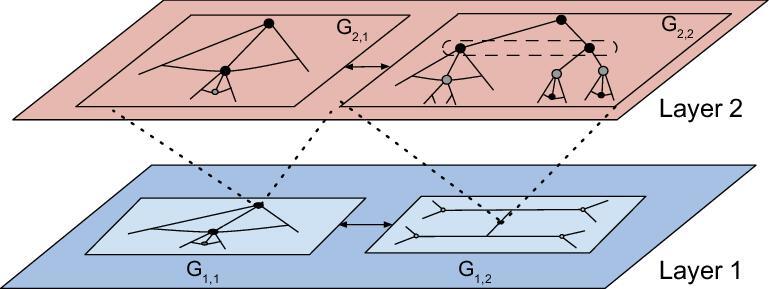
Games-in-games framework for secure and resilient control of multi-layer multi-agent systems. The control of each agent considers the behaviors of the agents at the same layer and the ones at the other layer. Furthermore, the agents also learn and respond to the unanticipated events, such as natural disruptions and adversarial attacks, at each step of decision making.

Given the cyber security strategy pair (*a, l*), the goal of robust and resilient control is to design a controller that minimizes the performance loss due to the attack, which is measured by the shaded area in Fig. 5. This design problem can be captured by an *H*^∞^ control problem with the performance index given by the expected cost over the statistics of θ:

(3) \begin{eqnarray*} \inf _{\bf u} \sup _{\bf w} \ J_P({\bf u, w}): = \mathbb {E}_\theta \lbrace q_{f}({\bf x}(t_{f}); \theta (t_{f})) \\ +\, \displaystyle \int _{t_{0}}^{t_{f}} g(t, {\bf x}(t), {\bf u}(t), {\bf w}(t); \theta (t))dt \rbrace, \end{eqnarray*}

where *q*_*f*_ is continuous in **x**, and *g* is jointly continuous in (*t, x, u, w*). In the infinite-horizon case, *q*_*f*_ is dropped out, and *t*_*f*_ → ∞. The *H*^∞^-optimal control problem in the time domain is in fact a minimax optimization problem and hence a zero-sum differential game, where the controller **u** can be viewed as the minimizing player and the disturbance **w** as the maximizing player [11,23]. The game (3) is referred to as the physical system game (PSG), and its solution is characterized by SPE. This framework enables the design of robustness and resilience within the same model, and takes into account the security vulnerabilities from the cyber systems. A large number of papers [18,24,40–44,49] has adopted the idea of deploying dynamic games for the security and resilience of modern control systems with interdependent cyber and physical layers. Many physical systems, including multibody robotic systems, power network systems and water distribution systems, are governed by differential-algebraic equations. To solve game (3) with differential-algebraic equations, one can refer to Ref. [58]. For specific systems, one can adopt specific models including Markov decision processes, difference equations and partial differential equations, to describe the dynamics in the physical layer and the cyber layer. The choice of dynamic models is dependent on the systems one is looking into.

#### Cyber-physical co-design and tradeoffs among robustness, security and resilience

The cyber-physical nature of modern control systems requires a cross-layer approach for designing secure and resilient systems. Independent designs of the cyber and the physical layers of the system without knowing their interdependencies often lead to unintended performance degradation. Thus, a co-design process that coordinates between cyber and physical layers of the system is pivotal for the control system. As illustrated in Fig. 3, the two design processes can be composed together and reach an iterative process for cyber-physical co-design. The resilient control design pair $(\mathbf {u}, \mathbf {w})$ will be used by the cyber system for the design of defense strategy pair (*a, l*), and likewise, the strategy pair (*a, l*) is also used by the physical system for the design of the control pair $(\mathbf {u},\mathbf {w})$. The coupled system leads to a holistic design framework that enables robust, secure and resilient design of infrastructural systems. The fundamental tradeoffs between robustness, security and resilience can be quantitatively analyzed and designed:

(i) Tradeoff between robustness and resilience. Perfect robustness of control systems is not achievable for all types of disturbances and events. However, resilience can be used as a post-event measure to recover the system from the impact of the disturbances and events that are not accounted for in the model. This tradeoff is captured by PSG for the security-aware resilient system design.
(ii) Tradeoff between security and resilience. Perfect security that is capable of defending against all types of attacks is not realistic for control systems. However, the resilient cyber systems can be designed to quickly bring a compromised state to their normal operations. This tradeoff is captured by the cyber system game (CSG) for the impact-aware proactive cyber defense.
(iii) Tradeoff between robustness and security. The two tradeoffs above lead to a relation between the robustness of the physical system and the security of the cyber system. The high demand for robustness requires a strong level of security. Given limited resources, they cannot be achieved at the same time. This tradeoff is captured by the coupled PSG and CSG frameworks.

#### Human factors in control systems

The human factors arise from the interactions between the control systems with the supervisory layer and the management layer. The supervisory layer provides human–machine interactions and coordinates and manages lower layers through centralized command and control as illustrated in Fig. 1. The behaviors of human designers and human operators are often less predictable and difficult to describe. They are often viewed as the weakest link in the control system. Attackers can leverage human vulnerabilities to enter and penetrate the multi-layer control system network. For example, in the Stuxnet attack [5,6], the maintenance engineer connected an infected USB to his maintenance laptop from which the malware entered the private network and caused a SCADA infection. And in the Maroochy breach [59], a former employee installed a SCADA configuration program on his own laptop and took control of 150 sewage pumping stations resulting in severe environmental damage.

The human factors have been studied extensively in the game theory literature with the objective to describe the cognitive, memory, computational and psychological aspects of the human decision-making process [60,61]. One important area of research is the bounded rationality which captures the behavioral and imperfect decision-making of humans. Several elements in the game-theoretic framework in CSG and PSG can be revised to capture human errors in decision making due to limited memory, attention or reasoning power. For example, by leveraging the concept of hyperbolic discounting, we can model the time-inconsistent human preferences, which have been demonstrated [62] to show that the human makes irrational choices at different times. Prospect theory [63,64] incorporates loss-aversion in human decisions and differentiates the perception of losses from the utility of the gains. It can be used to extend the risk-neutral decision-making in CSG and PSG to their risk-averse counterparts to understand the consequence of the cognitive bias in the decision-making.

Attention is another important human factor that can be incorporated in the decision making to capture the limited cognition of the human when they make online decisions [65]. Authors in Ref. [66] have presented an attention-constrained risk analysis model to assess risks over interdependent risk networks. The management layer at the highest echelon deals with social and economic issues, such as market regulation, pricing and incentives. Players in this layer deal with socio-economic issues involving many stakeholders related to the control systems and make service-level contracts to reduce cyber-physical risks. For example, cyber insurance is an example of financial products to transfer the risk from the control system and mitigate the losses due to cyber threats. Authors in Refs [67,68] have designed incentive-compatible attack-aware cyber insurance policies to maximize the social welfare and alleviate the impact of moral hazard. In Ref. [69], the authors have designed service contracts for security services in the cloud-enabled autonomous systems.

As the modern control system scales to billions of connected devices and is increasingly complex, it is not always possible for an entity to own and manage all cyber and physical components of the control system. For example, in cloud-enabled systems [34,70,71], smart homeowners use the services provided by the cloud service provider (SP) who fuses data and optimizes control decisions for real-time systems. Small business owners may not own the sensors but subscribe to service providers (SPs) who collect data that allow users to develop control system applications instead of making a costly investment in their own sensing infrastructure [72].

The decentralized ownership and the provision of control system services provide an effective sharing and utilization of the resources of computational, communication and sensing infrastructures. In this paradigm, the SP owns the cyber infrastructure and determines defense strategy *l* while the user owns the physical infrastructure and designs control **u**. However, a user cannot directly control or manage the security risk. If the SP is negligent in assuring cybersecurity, then users who rely on these services will be subject to high-security risks. It is essential to develop appropriate incentive-compatible service mechanisms for the SPs to offer high quality of services (QoS) while making efforts to mitigate security risks at the service level of control systems. SPs should be incentivized to deploy adequate security mechanisms to ensure the reliability and the dependability of the services for control system users. It not only enables the implementation and investment of security but also prevents the cyber risks from further propagating at the socio-economic scale.

Challenges in the design of the cyber-physical contract come from incomplete information and adversarial behaviors. The incomplete information can arise from the hidden type and the hidden action of the SP. In Refs [71,73], the authors have used contract design principles to develop a holistic incentive-compatible and cost-efficient security-aware service mechanism for real-time operation of cloud-enabled Internet of Controlled Things (IoCTs) under APTs.

## RECENT ADVANCES

With the hierarchical perspective toward robust, secure and resilient control systems, this section aims to introduce several recent dynamic applications to cross-layer control design in adversarial environments. Game-theoretic approaches have been natural frameworks to model conflicts between an attacker and a defender in various scenarios at the communication and networking layers including intrusion detection [44,74–78], jamming and eavesdropping [79–82], and honeypot/deception [83–87]. Apart from those at the cyber layers, game theory has also successfully addressed risk management [67,88] and security investment problems [66,89] at the human layers and the problem of adversarial consensus [90–92] and resilient infrastructures [93–98] at the control layers.

This section presents three quintessential research problems that represent three distinct directions where dynamic game approaches can be useful to bridge between multiple research areas and make significant contributions to the design of modern control systems. The first one leverages a moving-horizon dynamic game technique to secure the heterogeneous autonomous vehicles and enable self-healing after attacks. The second research direction investigates an impact-aware multi-stage cyber deception game where the defender proactively deters the stealthy APT attacks from reaching the critical asset of industrial control systems. Adversarial and defensive deceptions across the entire intrusion process introduce the games of incomplete information, thus both players need to make judicious actions under persistent uncertainty. The third direction focuses on the risk management of networked systems by incentivizing agents to comply with security guidelines with maximum effort.

### Games for secure control of heterogeneous autonomous systems

Multi-layer networks or network-of-networks have been seen in a number of critical applications, such as energy and water networks [99], power and transportation networks [100] and multi-layer robotic systems [49]. Traditional defensive mechanisms for single networked systems are no longer sufficient for this network-of-networks paradigm. To design secure and resilient control strategies for the multi-layer autonomous systems, it is imperative to analyze three types of games resulting from the strategic interactions: (i) interactions among agents in individual network layers, (ii) interactions between agents from different layers, and (iii) interactions between agents and adversaries. To address this challenge, the authors in Refs [39,101] have proposed a ‘games-in-games’ model which is able to understand the network performance, heterogeneous agents’ functionalities and the network operators’ decisions holistically.

For clarity, a pictorial illustration of the games-in-games framework is shown in Fig. 5. The system composes two layers of networks. In each sub-layer, agents make decisions based on not only the behaviors of the agents at the same layer but the ones at the other layer. At each step of decision making, the agents also learn and respond to the unanticipated events in an agile fashion, such as natural disruptions and adversarial attacks. Leveraging the framework, one can compose the distinct games together to obtain the Gestalt Nash equilibrium (GNE) [66,70]. The GNE describes an equilibrium solution concept at which no agent has incentives to deviate away from not only each modular game, which captures the local agent–agent level interactions, but also the integrated game, which considers the global system–system level interactions.

Based on Ref. [101], we next present an example of controlling two-layer mobile autonomous systems in the adversarial environment. There are three players in the game: two network operators and an attacker. The focused objective in Ref. [101] is the algebraic connectivity of the global network. This performance metric quantifies how well connected is the network. If the algebraic connectivity is zero, then the system is disconnected, indicating that at least one agent in the network is separated from the rest of the agents. Furthermore, a larger value of algebraic connectivity leads to faster information spreading between agents, resulting in a higher level of situational awareness. Thus, maximizing the algebraic connectivity is important for the operators, especially when the autonomous systems are adopted in the mission critical applications in the adversarial environment. To this end, the attacker’s problem at time *k* is formulated as follows:

(4) \begin{equation*} \mathcal {Q}_A^k:\ \ \ \min _{e}\ \lambda _2(e,\mathbf {x}(k)), \end{equation*}

where $\lambda _2(e,\mathbf {x}(k))$ is the connectivity of the global network, with *e* representing the attacker’s strategy and $\mathbf {x}(k):=[\mathbf {x}_1(k);\mathbf {x}_2(k)]$ the network configuration at time *k*. On the other hand, the network operator γ’s problem is, for γ ∈ {1, 2}:

(5) \begin{eqnarray*} \mathcal {Q}_\gamma ^k:\ \max _{\mathbf {x}_\gamma ({k+c_\gamma })}\ \min _{e}\ \lambda _2(e,\mathbf {x}({k+c_\gamma })) \nonumber\\ \mathrm{s.t.}\ \mathrm{physical\ dynamics\ of\ autonomous\ systems}, \end{eqnarray*}

where $\mathbf {x}_\gamma ({k+c_\gamma })$ is the configuration of the mobile network controlled by operator γ at time *k* + *c*_γ_ and *c*_γ_ is a positive integer indicating update frequency. Note that the network operator’s problem falls into the general framework formulated in the earlier section on ‘Dynamic games for robustness, security and resilience’, where the dynamics of autonomous systems can be captured by Eqs (1) and (2), and the parameter θ is regarded as fixed. The objective function of the operator remains to be algebraic connectivity at every time step, which is different from the one in Eq. (3). However, the dynamic feature is also incorporated in this example scenario, as the operator needs to reconfigure the autonomous network through considering the adversarial behavior at each time step. The proposed games-in-games model also extends the single layer attacker–defender framework in the section ‘The cyber-physical-human system framework’ to address the secure control of heterogeneous autonomous networks. Specifically, each network operator needs to prepare for the worst case attacks (Stackelberg game) as well as the action taken by the other operator (Nash game) during the network reconfiguration.

**Figure 6.**
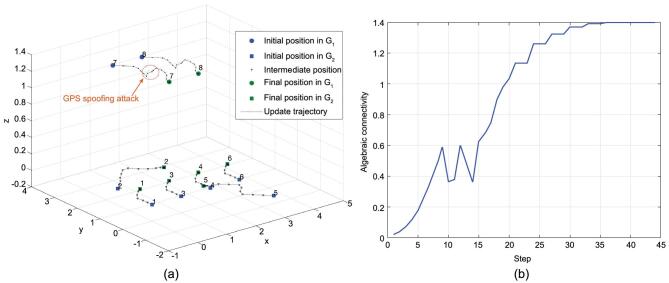
(a) The dynamic and secure configuration of a two-layer robotic network. The GPS spoofing attack is introduced at time step 9 and lasts for five steps. (b) The corresponding network connectivity.

This games-in-games framework has been corroborated to be effective in obtaining the self-adaptability, self-healing and agile resilience of heterogeneous autonomous systems. In the Internet of battlefield things, the unmanned ground vehicle network coordinates its actions with the unmanned aerial vehicle network and the soldier network to achieve a highly connected global network [102]. The designed decentralized algorithm in Ref. [101] yields an intelligent control of each agent to respond to others to optimize real-time network connectivity under adversaries. Figure 6 shows the results of a two-layer autonomous system on the battlefield where two operators prepare for potential jamming attacks. Furthermore, the agents can respond to the spoofing attack quickly which shows the agile resilience of the control strategy. The developed games-in-games model can be further extended to address the ‘mosaic control design’ as the framework

provides built-in security and resilience for each component in the system, which guarantees the performance of the integrated system.

### Multi-stage Bayesian games: security under adversarial and defensive deception

**Figure 7.**
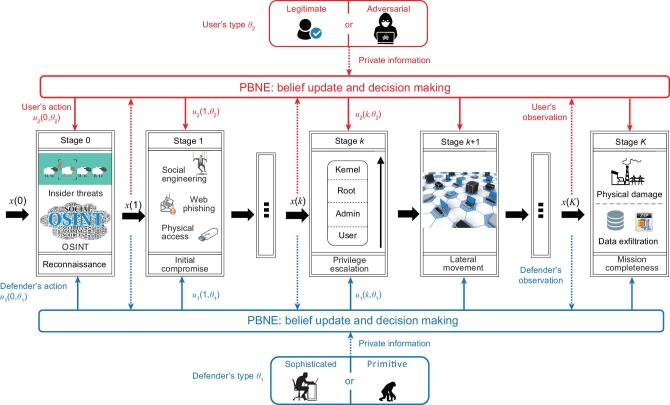
A block diagram of the proposed proactive defense-in-depth paradigm against multi-stage stealthy APTs. As denoted in black, each stage describes a local interaction between the user and the defender where the outcome leads to the next stage of interactions. Dashed arrows represent the information available to each player, which can be used to update the belief and decide the cross-stage behavioral strategy based on the PBNE. Then, each player takes an action at each stage according to the strategy, as denoted in solid arrows.

APT attacks originated from a cyber network (the middle layer of Fig. 1) can stealthily escalate privilege, move laterally and lead to damage in the physical control system (the bottom layer of Fig. 1). The entire intrusion process can be divided into multiple phases in sequence, as denoted by the black boxes in the middle layer of Fig. 7. Each phase serves as a stepping stone for the next phase and plays an indispensable role in the success of APTs. Based on the multi-stage and stealthy characteristics of APTs, Ref. [103] has suggested a ‘Defense-in-Depth’ (DiD) paradigm to counter them ‘proactively’. DiD as the first aspect means that a control system defender should adopt defensive countermeasures at all phases of APTs and holistically consider interconnections and interdependencies among these stages. For example, a privilege restriction at the escalation phase can result in a failure or an additional cost for the APT attacker to take control of the targeted sensor at the final stage. Proactive actions and precautions as the second aspect mean that the defender needs to act before an attack is revealed. On one hand, these precautions can mitigate the loss induced by the APT attack at the final phase and deter attacks at their early stages. On the other hand, they can also impair the user experience and reduce the utility of legitimate users. Hence, the defender has to take judicious actions at each stage to balance usability versus security.

The lower and upper layers of Fig. 7 illustrate a *K*-stage strategic interaction between the proactive defender and the user in blue and red, respectively. The type of a user θ_2_ can be either adversarial or legitimate. Since an APT attacker can pretend to be a legitimate user throughout stages, the defender does not know the user’s type. The defender can observe suspicious user actions at each stage. However, these suspicious actions do not directly reveal the user’s type because a legitimate user may also take them. For example, both the Tor network connection [104] and the code obfuscation [74] can be used legitimately or illegally. Similarly, a defender can also be classified into different types θ_1_ based on factors such as their level of security awareness, detection techniques they have adopted, and the completeness of their virus signature database. To tilt the information asymmetry that the user has a private type, the defender can also introduce defensive deception and make their type unknown to the user. The defender takes proactive actions at each stage and the user can observe them at the

next stage. Therefore, each stage describes a local interaction between the attacker and the defender (a two-player game) where the outcome leads to the next stage of interactions. The system state transition is described by a controlled Markov game (6) and belongs to the dynamics Eq.(1) with a static θ ≔ {θ_1_, θ_2_}, i.e. for *k* = 0, 1, ⋅⋅⋅, *K* − 1,

(6) \begin{eqnarray*} \mathbf {x}(k+1) &=& f(\mathbf {x}(k),\lbrace u_1(k,\theta _1),u_2(k,\theta _2)\rbrace ), \nonumber\\ \mathbf {x}(0) &=& x_0, \end{eqnarray*}

where *u*_1_(*k*, θ_1_) and *u*_2_(*k*, θ_2_) represent the action of the defender and the user at stage *k*, respectively. Participants receive different stage utilities from each local interaction (a non-zero-sum discrete counterpart of Eq. (3)) and each player aims to find a behavioral strategy for this dynamic game to maximize his expected utility accumulated over *K* stages. The behavioral strategy means that each player needs to decide which action to take or take an action with what probability based on the available information at each stage *k* ∈ {0, 1, ⋅⋅⋅, *K*}, i.e. $I_i^k:=\lbrace x(0),\cdots ,x(k-1),\theta _i\rbrace , \forall i\in \lbrace 1,2\rbrace$. Each player *i* introduces a belief $b_i^k$ at each stage *k* to quantify the uncertainty of the opponent’s type and adopts the Bayesian update in (7) to correlate the information revealed at each stage and reduce the type uncertainty, i.e. for *i, j* ∈ {1, 2}, *j* ≠ *i*,

(7) \begin{eqnarray*} && b^k_i(\theta _{j} | I_i^{k+1}) \nonumber\\ && =\, \frac{\Pr (x(k+1)| x(k),\theta _1,\theta _2)b_i^{k}(\theta _{j}|I_i^{k})}{\sum _{\bar{\theta }_{j}\in \Theta _j} \Pr (x(k+1)| x(k),\theta _i,\bar{\theta }_{j})b_i^{k}(\bar{\theta }_{j}|I_i^{k})}. \nonumber\\ \end{eqnarray*}

The solution concept of Perfect Bayesian Nash Equilibrium (PBNE) is introduced where ‘perfect’ captures the cross-stage cumulative utility, ‘Bayesian’ captures the type uncertainty, and ‘Nash Equilibrium’ captures the strategic interaction between two players. The PBNE provides a creditable predication of both players’ behaviors over *K* stages because no players benefit from unilateral deviations at the equilibrium. The term $\Pr (x(k+1)| x(k),\theta _1,\theta _2)$ in the forward belief update Eq. (7) depends on the behavioral strategy of both players. In the meantime, the strategy computation in a backward fashion depends on the belief. To solve this coupling, Ref. [103] has proposed a sequence of nested algorithms and Refs [31,105] have adopted conjugate priors to enable parametric learning. The authors in Ref. [103] have also provided an elaborate case study of APT attacks on the Tennessee Eastman process (a specific example of Eqs (1) and (2)) and obtained the following insights. First, one ounce of proactive actions when the attack remains ‘under the radar’ is worth a pound of post-attack response. Second, the online learning capability of the defender reveals hidden information from observable behaviors and threatens the stealthy attacker to take more conservative actions. Third, defensive deception introduces uncertainty to attackers, increases their learning costs, and hence reduces the probability of successful attacks.

#### Comparison and discussion

To provide a broad view of applying dynamic games for APT defense, we review other dynamic game models for APT detection and response and compare them with the benchmark model introduced above. Although APTs are stealthy and customized, their interactions with the system introduce information flows of data- and control- commands. An alternative perspective for APT defense is to respond to and mitigate the effect of APTs under perfect or imperfect APT detection. The authors in Ref. [29] have identified a sequence of heterogeneous game phases, i.e. a static Bayesian game for spear phishing, a nested game for penetration, and a finite zero-sum game for the final stage of physical-layer infrastructure protection. On the other hand, Ref. [106] has proposed a differential game approach to repair the system efficiently from an APT incident. Both frameworks consider the dynamic feature of APTs, yet they both assume complete information, which relies on a perfect detection of APTs. The FlipIt game [107] considers the APT response problem under imperfect detection, i.e. the defender does not know when stealthy APT attackers take control of the system until he takes a takeover action with additional cost. The FlipIt has described a high-level abstraction of the attackers' stealthy takeover behavior to understand optimal timing for resource allocations. On the other hand, in Ref. [108], the model is based on a sequence of nested finite two-person zero-sum games, in which the APT is modeled as the attempt to get through multiple protective shells of a system towards conquering the target located in the center of the infrastructure. Besides, the model proposed in Ref. [103] provides a finer-grained model that can capture heterogeneous adversarial and defensive behaviors at multiple stages, allowing the prediction of attack moves and the estimation of losses using the equilibrium analysis.

### Dynamic games for risk management of networked systems

Game theory is widely adopted in the risk management of complex engineering systems [66,67]. Mitigating the risk of multi-agent systems is critical for their secure and efficient operations. However, due to complex interdependencies between nodes and the fast-evolving nature of threats, controlling the risks of multi-agent systems is not a trivial task and requires expert knowledge. Hence, one approach for the system owners is to delegate tasks of risk mitigation to security professionals, creating security as a service paradigm [73].

**Figure 8.**
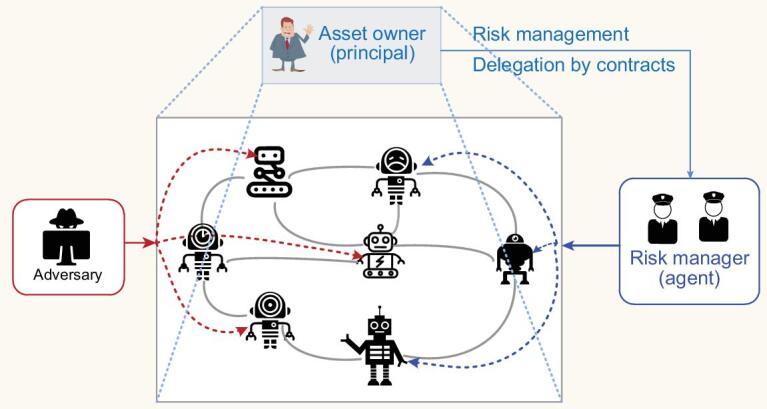
Risk management of a networked system through dynamic contracts. The asset owner (principal) delegates the risk management tasks to security professional (agent) by designing a contract that specifies the dynamic remuneration schemes. The agent’s effort is hidden to the principal. The amount of remuneration depends on the observed risk of the system. The contract mechanism design can be formulated as a stochastic Stackelberg differential game under non-standard information.

As shown in Fig. 8, the owner can be seen as a principal who employs a security professional to fulfill risk management tasks, and the security professional (risk manager) can be regarded as an agent whose efforts are dynamically compensated by the principal. This type of two-sided service relationship can be captured by a principal–agent framework. One unique feature of the framework is that the principal cannot directly observe the efforts adopted by the agent. Thus, the principal needs to design a contract that specifies the compensation rules only based on observable risk outcomes. Specifically, the cyber risk evolution can be described by the following dynamic systems (which belongs to the general dynamics Eq. (1)):

(8) \begin{eqnarray*} d\mathbf {x}(t) &=& f(\mathbf {x}(t),\mathbf {u}(t),t)dt + \Sigma (\mathbf {x}(t),t)d\mathbf {b}(t), \nonumber\\ \mathbf {x}(0) &=& x_0, \end{eqnarray*}

where $f:\mathbb {R}^N\times \mathbb {R}_+^N \times [0,T]\rightarrow \mathbb {R}^N$, $\Sigma :\mathbb {R}^N \times [0,T]\rightarrow \mathbb {R}^N$ with $\mathbf {x}(t)\in \mathbb {R}^N$ represents the risk of nodes in the system, $\mathbf {u}(t)\in \mathbb {R}_+^N$ the hidden effort of the agent, $\mathbf {b}(t)$ is an *N*-dimensional standard Brownian motion, and *x*_0_ is a known *N*-dimensional constant vector indicating the initial risk. The dynamic contract designed by the principal is *p*(*t*), *t* ∈ [0, *T*], reflecting the payment delivered to the agent at time *t*. First, the principal’s goal is to minimize the risk $\mathbf {x}(t)$ by providing an appropriate amount of incentives *p*(*t*) over the time horizon.

Second, the contract should capture the agent’s behavior including the incentive compatibility (IC) and the individual rationality (IR). The principal’s cost function is

(9) \begin{equation*} J_P(\lbrace p(t)\rbrace _{0\le t\le T}) = \mathbb {E}\int _0^T f_P(t,\mathbf {x}(t),p(t))dt, \end{equation*}

and the agent’s cost function is

(10) \begin{eqnarray*} && J_A\left(\lbrace \mathbf {u}(t)\rbrace _{0\le t\le T};\lbrace p(t)\rbrace _{0\le t\le T}\right)\\ && = \mathbb {E}\int _0^T f_A(t,p(t),\mathbf {u}(t)) dt, \end{eqnarray*}

where $\mathbb {E}$ is the expectation operator, and *f*_*P*_ and *f*_*A*_ are the running costs of two players. Furthermore, the IC constraint is $J_A\left(\lbrace \mathbf {u}(t)^*\rbrace _{0\le t\le T};\lbrace p(t)\rbrace _{0\le t\le T}\right) \le J_A\left(\lbrace \mathbf {u}(t)\rbrace _{0\le t\le T};\lbrace p(t)\rbrace _{0\le t\le T}\right),\ \forall \mathbf {u}(t),\ t\in [0,T],$ and the IR constraint is $\inf _{\mathbf {u}(t)} J_A\left(\lbrace \mathbf {u}(t)\rbrace _{0\le t\le T};\lbrace p(t)\rbrace _{0\le t\le T}\right) \le \underline{J}_A,$ where $\underline{J}_A$ is a predetermined non-positive constant.

This contract design for a risk management problem can be formulated as a ‘stochastic Stackelberg differential game under non-standard information’. To design the optimal contract, the authors in Refs [88,109] have developed a three-step approach including the estimation, verification and control phases, which transformed the principal’s non-classical control problem into a standard stochastic control program. For example, when the dynamics in Eq. (8) admit a linear form and the players’ cost functions are quadratic, then the optimal contract can be obtained through solving a matrix Riccati equation [88]. Under mild conditions on the structure of cost functions of two players, the authors have revealed a ‘separation principle’ where the estimation and control phases can be addressed separately. The authors have also discovered a ‘certainty equivalence principle’ for a class of dynamic mechanism design problems where the contracts designed under incomplete case and full information scenario (the principal can directly observe the agent’s action) coincide. The contract mechanism has been corroborated effective in mitigating the risks.

The developed framework for risk management can be applied broadly, such as industrial control systems, enterprise networks, and critical infrastructures. Furthermore, the dynamic mechanism design problem can be extended extensively, which is of great interest to the control community. For example, the underlying system could have jump parameters, the risk could be governed by mean-field dynamics in large networks, the risk cannot be directly observable to the players, and the risk observation is intermittent, etc.

## CONCLUSION AND FUTURE DEVELOPMENT

In this review, we have discussed recent advances and applications of dynamic games to the robust, secure and resilient design of modern control systems. We have introduced the hierarchical structure of modern control systems, offering a holistic view of control systems that leads to an integrated dynamic game framework. The dynamic games approach has successfully captured the multi-layer cyber, physical and human interactions in control systems as well as their behaviors in adversarial environments. The game-theoretic modeling has provided a fundamental understanding of the tradeoffs among robustness, security and resilience, leading to a new system science and design paradigm.

The application of dynamic games to control systems is still in its infancy despite a rich literature in game and control theory. The bridging between these two fields would require addressing many research challenges. Computational complexity is one important research direction. Analysis of large-scale game-theoretic models is often difficult. It would be essential to develop efficient algorithms to compose distinct models, compute equilibrium solutions, and solve mechanism design problems. These tools would lead to the core of the next-generation control system technologies that have the capabilities of automated defense, self-organizing and fast recovery.

Another key challenge arises from dealing with human factors at the supervisory and management layers. It has been observed that many security breaches are due to human cognitive errors, limited reasoning capabilities, and mismatched perception of risk. Integrating human modeling into control systems is critical to enable a scientific framework for human-centered design. Recent advances in behavioral game theory and epistemic game theory have laid necessary theoretical foundations for the modeling of bounded rationality and human behaviors. Hence game theory provides an unprecedented opportunity to understand human factors in control systems by bridging game theory and control system theory.
